# The legal and ethical considerations in cross-border telesurgical procedures

**DOI:** 10.1097/MS9.0000000000003344

**Published:** 2025-05-12

**Authors:** Chukwuka Elendu, Dependable C. Amaechi, Tochi C. Elendu, Emmanuel C. Amaechi, Ijeoma D. Elendu, Olutomiwa A. Omokore, Nwamaka C. Onubogu, Janet C. Omeludike, Eunice T. Aregbesola, Oluwagbemiga O. Fajimi, Omoyelemi F. Idowu, Sopuruchukwu L. Emechebe, Mbanefo C. Uyanwune, Johnson Yonni

**Affiliations:** a Federal University Teaching Hospital, Owerri, Nigeria; b Igbinedion University, Okada, Nigeria; c Imo State University, Owerri, Nigeria; d Madonna University, Elele, Nigeria; e Babcock University Teaching Hospital, Ilishan-Remo, Nigeria; f Abia State University, Uturu, Nigeria; g University of Chester, Chester, UK; h University of Missouri, Columbia, South Carolina; i Canterbury Christ Church University, Canterbury, England; j Prime Specialist Hospital, Lagos, Nigeria; k Nnamdi Azikiwe University, Awka, Nigeria; l Bingham University, Nasarawa, Nigeria

**Keywords:** 5G networks, artificial intelligence, cross-border healthcare, robotic surgery, telesurgery

## Abstract

Telesurgery, or remote surgery, represents a transformative fusion of medicine and technology, enabling surgeons to perform procedures on patients located miles away using robotic systems and advanced telecommunications. However, its widespread adoption remains limited, with fewer than 50 documented fully remote telesurgical procedures in the past two decades. While robotic-assisted surgery is increasingly common – accounting for over 1.2 million procedures in 2019 – true cross-border telesurgery is rare due to technological, legal, and ethical barriers. The lack of a unified regulatory framework presents jurisdiction, licensing, liability, and data security challenges, particularly when procedures span international borders. The absence of standardized legal mechanisms creates uncertainty, especially in surgical complications or malpractice claims. A global regulatory framework should address these challenges, incorporating mutual recognition of medical licenses, standardized liability agreements, and uniform data protection protocols aligned with General Data Protection Regulation and Health Insurance Portability and Accountability Act. Additionally, real-time 5G-enabled monitoring systems could mitigate latency issues, reducing the risk of surgical errors due to connectivity disruptions. Ethically, telesurgery raises concerns regarding informed consent, equitable access, and accountability. Language barriers, differing cultural attitudes toward robotic surgery, and disparities in healthcare infrastructure complicate ethical oversight. Establishing an international telesurgical ethics board could ensure adherence to standardized consent procedures and promote equitable access through global partnerships. Investment in telesurgical training programs and AI-driven risk mitigation strategies could enhance patient safety. While technological advancements will continue to drive telesurgery’s growth, addressing these legal and ethical considerations through harmonized global regulations and strategic policy interventions is crucial for sustainable and equitable integration into modern healthcare.

## Introduction and background

As telesurgery continues to evolve, its expansion beyond national borders presents an urgent need for thorough legal and ethical frameworks. While the technology enables seamless surgical collaboration between geographically distant locations, the absence of harmonized policies has led to uncertainties regarding professional accountability, patient rights, and the operational feasibility of such procedures on a global scale^[1–3]^. Unlike conventional surgical practices, where legal and ethical responsibilities are clearly defined within national healthcare systems, cross-border telesurgery introduces novel complexities that demand innovative governance solutions. Despite the increasing adoption of robotic-assisted surgery – accounting for over 1.2 million procedures in 2019 – true telesurgery remains exceedingly rare, with fewer than 50 fully remote telesurgical procedures documented in the past two decades^[1,2]^. While experimental successes, such as the 2001 Lindbergh operation, have demonstrated the feasibility of long-distance robotic surgery, widespread clinical implementation has been limited due to technological, legal, and ethical constraints^[3]^. The scarcity of real-world telesurgical procedures underscores the gap between technological potential and practical application, necessitating urgent regulatory and policy adaptations to support its responsible growth.

**HIGHLIGHTS**

- Legal and ethical challenges shape cross-border telesurgery regulations.
- Patient safety, cybersecurity, and liability remain key concerns in telesurgery.
- Data privacy, licensure, and jurisdiction add complexity to cross-border telesurgery.

Furthermore, the reliance on sophisticated robotic surgical platforms and real-time digital communication necessitates a multidisciplinary approach to regulation. The interplay between medical ethics, international law, cybersecurity, and telecommunication standards underscores the multifaceted nature of cross-border telesurgery^[4–6]^. Addressing these challenges requires a legal response and proactive engagement from international health organizations, policymakers, and technology developers to ensure that telesurgical interventions are safe and ethically sound. Another pivotal consideration is interdisciplinary collaboration’s role in shaping telesurgery’s future. Integrating artificial intelligence (AI)-driven decision support systems, blockchain-based patient data management, and 5G-enabled ultralow latency communication is revolutionizing remote surgical capabilities^[7]^. However, these advancements must be accompanied by policy adaptations safeguarding patient welfare while fostering innovation. Establishing clear guidelines for data ownership, cross-border liability frameworks, and the standardization of telesurgical training programs can help mitigate risks associated with the widespread adoption of this technology^[8–10]^. Given the current regulatory gaps, our review underscores the need for globally coordinated efforts to establish standardized legal and ethical principles for cross-border telesurgery. This requires legislative initiatives and capacity-building programs that empower healthcare institutions worldwide to integrate telesurgical practices responsibly. By exploring potential frameworks for international cooperation, our paper aims to contribute to ongoing discussions on the sustainable and ethical expansion of telesurgery across borders.

## Data collection

A literature review was conducted using PubMed, Scopus, and Google Scholar databases, focusing on articles published from 2000 to 2024. The keywords used included “telesurgery,” “cross-border healthcare,” “legal considerations,” “ethical challenges,” and “remote surgery.” Articles were included if they addressed telesurgery’s legal, ethical, or regulatory aspects, emphasizing cross-border scenarios. The study design is descriptive and analytical, aiming to synthesize existing knowledge and identify gaps in the current regulatory and ethical frameworks. Relevant case studies, including the Lindbergh Operation and other documented telesurgical procedures, were reviewed to illustrate practical challenges and solutions. The literature review included peer-reviewed journal articles, official regulatory documents, and expert consensus reports. Articles published in English that provided specific insights into cross-border telesurgery were prioritized. Exclusion criteria involved studies focused solely on technological or clinical aspects of telesurgery without addressing legal or ethical dimensions. Data extracted from the literature were analyzed thematically to identify recurring challenges such as jurisdictional ambiguities, licensing issues, data protection concerns, and equity in access. Ethical considerations, including patient consent, safety, and trust, were also examined in detail. This analysis informed the recommendations for creating standardized global policies and ethical guidelines for cross-border telesurgery.

## Current progress and potential of telesurgery

The evolution of telesurgery began with the development of robotic systems such as the da Vinci Surgical System, which allowed surgeons to perform minimally invasive surgeries with high precision. Although initially operated locally, these systems paved the way for cross-border applications^[1]^. The landmark Lindbergh Operation in 2001 marked the first fully transatlantic telesurgery, wherein a surgeon in New York successfully performed a cholecystectomy on a patient in Strasbourg, France. This milestone demonstrated the feasibility of remote surgery, even in high-stakes scenarios, provided the infrastructure supported low-latency, high-bandwidth communication^[2]^. Continuous advancements in robotic platforms, imaging technology, and telecommunication infrastructure drive current progress in telesurgery. Robotic systems such as the Zeus Robotic Surgical System and the da Vinci Xi platform now integrate features like 3D imaging, AI-powered decision support, and ergonomic controls, enabling surgeons to execute intricate maneuvers with millimeter precision.

Additionally, enhanced haptic feedback in some systems provides tactile sensations, bridging the sensory gap that often exists in remote operations^[3]^. The role of 5G networks in telesurgery cannot be overstated. High-speed, low-latency networks ensure real-time communication between the surgeon and the robotic system. Studies have shown that latency beyond 200 milliseconds can significantly compromise surgical performance and patient safety^[4]^. With the rollout of 5G technology globally, telesurgery is becoming increasingly feasible in remote or underserved regions, eliminating geographical disparities in access to specialized care. In 2020, Chinese surgeons conducted a successful telesurgery operation using 5G networks to treat a patient with Parkinson’s disease, highlighting the transformative potential of such infrastructure^[5]^. AI and machine learning (ML) have also enhanced the potential of telesurgery. AI algorithms analyze vast amounts of surgical data to provide intraoperative guidance, predict complications, and recommend optimal surgical approaches. AI-enabled telesurgery platforms are emerging as critical tools for improving efficiency and reducing errors, particularly in high-stakes environments^[6]^.

Furthermore, these platforms are being integrated with augmented reality and virtual reality technologies to provide surgeons with an immersive and interactive interface, further enhancing precision and situational awareness^[7]^. The applications of telesurgery extend far beyond routine surgical interventions. In military and disaster scenarios, telesurgery has proven invaluable in providing immediate surgical care to patients in inaccessible or dangerous locations. The use of robotic surgical systems in space exploration has also been explored. NASA, for example, has funded research into telesurgical capabilities for astronauts on long-duration missions, where access to terrestrial medical facilities is impossible^[8]^. Despite its significant progress, telesurgery faces several limitations. The high cost of robotic systems and infrastructure poses a significant barrier to widespread adoption, particularly in low- and middle-income countries.

## Legal considerations

**Figure 1.**
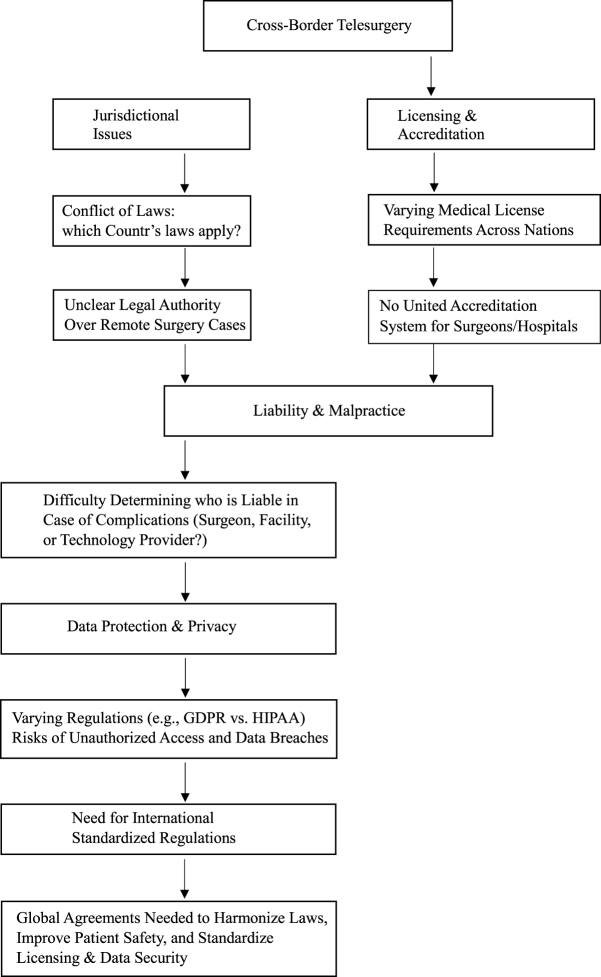
Major hurdles in cross-border telesurgery and their interconnected legal and ethical challenges. Our flowchart outlines the major hurdles in cross-border telesurgery and the interconnected legal and ethical challenges – source: Authors’ creations.

The emergence of telesurgical technology has redefined the boundaries of healthcare, enabling surgeons to perform complex procedures across geographical barriers. However, cross-border telesurgery introduces significant legal challenges that demand careful consideration (see Fig. 1)^[1–3]^. One of the most important legal challenges in cross-border telesurgery is the determination of jurisdiction. In traditional medical procedures, jurisdiction is typically established based on the healthcare provider’s location or the institution where the treatment is delivered. However, in telesurgery, the physical separation between the patient and the surgeon complicates this determination. For instance, if a surgeon in the United States operates on a patient in India using a robotic system, it is unclear whether U.S. or Indian laws govern the procedure^[4–6]^. This ambiguity can lead to disputes over liability, malpractice claims, and enforcement of legal judgments. A notable case illustrating this issue is the 2001 Lindbergh Operation, in which a French surgeon performed laparoscopic surgery on a patient in Strasbourg while operating from New York. Although no legal disputes arose, it highlighted the complexities of jurisdiction in cross-border telesurgery. Additionally, in the case of Gutnick v. Dow Jones (2002), though unrelated to telesurgery, the ruling established the principle that jurisdiction can be determined based on where the harm occurs, a precedent that may influence future telesurgical disputes^[2,3]^. To address this, international agreements or bilateral treaties may be required to clarify jurisdictional issues. Another critical consideration is the licensing and accreditation of surgeons and healthcare facilities. Medical practice regulations are highly localized, and a surgeon licensed in one country may not automatically be eligible to practice in another. Cross-border telesurgery raises questions about whether the surgeon should hold a license in the patient’s country, the country where the procedure is performed, or both. Failure to meet licensing requirements can lead to legal penalties and jeopardize the validity of malpractice insurance^[8–10]^. Additionally, healthcare facilities involved in telesurgery must meet international standards to ensure the safety and efficacy of the procedures. Accreditation by recognized bodies, such as the Joint Commission International (JCI), can help establish trust and compliance in cross-border telesurgical arrangements^[3,4]^. Liability and malpractice issues are also significant legal hurdles in telesurgery. Determining responsibility for adverse outcomes becomes more complex in cross-border settings. If a technical failure occurs during a procedure, it may be unclear whether the surgeon, the healthcare institution, or the technology provider should be held accountable. For example, in the case of Taylor v. Intuitive Surgical, Inc. (2017), a U.S. court ruled that a robotic surgery manufacturer was partially liable for inadequate surgeon training, raising questions about whether manufacturers bear responsibility in telesurgical malpractice claims. Similarly, in the case of Karam v. (2012), a patient sued a hospital after suffering complications from robotic-assisted surgery, highlighting liability concerns in telesurgery^[11,12]^. The manufacturer could be implicated if a robotic arm malfunctions due to software errors. Conversely, liability may fall on the surgeon or institution if the failure results from inadequate training or oversight. To mitigate these risks, detailed contracts specifying roles and responsibilities should be established between all parties involved. Moreover, comprehensive malpractice insurance policies that cover cross-border procedures are essential to protect surgeons and institutions from financial and reputational harm^[5,6]^. Data protection and privacy represent another area of legal concern. Telesurgery transmits sensitive patient information across borders, exposing it to potential breaches and unauthorized access. Compliance with data protection laws, such as the General Data Protection Regulation (GDPR) in Europe or the Health Insurance Portability and Accountability Act (HIPAA) in the United States, is critical^[5,13,14]^. These laws mandate strict safeguards for storing, processing, and sharing patient data. However, disparities in data protection standards across countries can create legal conflicts. For instance, in Schrems II (2020), the Court of Justice of the European Union invalidated the Privacy Shield framework, ruling that U.S. data protection standards were inadequate under GDPR. This decision has significant implications for cross-border telesurgery, as patient data may be subject to differing levels of protection^[7]^. Blockchain technology offers a potential solution to these data protection challenges by enabling decentralized and secure patient record management. Utilizing blockchain-based smart contracts allows patient data to be encrypted and shared only with authorized parties while ensuring compliance with international privacy laws. Additionally, blockchain can enhance transparency and traceability in liability disputes by providing immutable records of surgical procedures, access logs, and clinical decisions^[7,8]^. In addition to these challenges, cross-border telesurgery must address contractual and intellectual property issues. Contracts between surgeons, institutions, and technology providers must define the scope of services, payment terms, dispute resolution mechanisms, and termination conditions^[15,16]^. These agreements must also account for intellectual property rights related to telesurgical technology, such as software, algorithms, and robotic systems. Disputes over intellectual property ownership can arise if multiple parties contribute to developing or customizing the technology. The legal battle between Intuitive Surgical and Johnson & Johnson over robotic-assisted surgery patents demonstrates the complexities of intellectual property in telesurgical technology. In another case, Medtronic v. Mazor Robotics (2018), intellectual property disputes arose over robotic surgical systems, further emphasizing the need for clear agreements. To minimize such conflicts, clear ownership and licensing agreements should be established at the outset^[9,10]^. Another consideration is the need for harmonized international regulations to facilitate cross-border telesurgery. No universal framework governs these procedures, resulting in a fragmented legal landscape. Countries vary significantly in their medical practice laws, liability frameworks, and data protection standards, creating barriers to the widespread adoption of telesurgery^[17–19]^. For example, while the United States has developed telemedicine guidelines under the Federation of State Medical Boards (FSMB), countries like India have only recently introduced telemedicine regulations under the Telemedicine Practice Guidelines (2020), leading to inconsistencies in implementation. AI can be crucial in harmonizing regulations by facilitating real-time compliance checks and automating jurisdictional assessments. AI-driven legal analysis tools can help identify discrepancies in licensing requirements and liability frameworks across countries, allowing stakeholders to make informed decisions when engaging in cross-border telesurgery^[5,6]^.

## Ethical considerations

While telesurgical innovation offers immense potential for improving access to specialized care, it also raises complex ethical issues that require careful analysis and resolution^[20–22]^. One of the most pressing ethical concerns is the issue of informed consent. In cross-border telesurgical procedures, obtaining valid and informed consent becomes more challenging due to language barriers, cultural differences, and limited direct interaction between the surgeon and the patient. AI-powered natural language processing tools can help bridge this gap by translating consent documents into multiple languages and ensuring clarity in patient communication. Furthermore, AI-driven decision support systems can assess patients’ comprehension of the procedure, mitigating risks related to miscommunication. Another ethical issue is equity in access to telesurgery services. While telesurgery promises to bridge the gap in healthcare access between rural and urban populations and between high-income and low-income countries, the reality often falls short of this ideal^[12]^. The high costs of telesurgical technologies, including robotic systems and stable internet connectivity, may limit access to affluent healthcare institutions or regions with better infrastructure. This raises questions about fairness and whether telesurgery may inadvertently deepen existing disparities in healthcare access. To address these disparities, policymakers should consider implementing subsidized programs that provide LMICs access to telesurgical technologies through public-private partnerships. Additionally, international collaborations between medical institutions in high-income and low-income countries could facilitate training programs, knowledge exchange, and shared infrastructure development. Governments and global health organizations should also advocate for the inclusion of telesurgical procedures in universal health coverage schemes, ensuring affordability for underserved populations^[3,4]^. Also, blockchain-based funding models, such as decentralized health insurance and crowdfunding platforms, could provide financial support for telesurgical services in low-resource settings, improving access and reducing disparities. The ethical obligation to ensure patient safety is closely related to equity. AI can enhance patient safety by continuously monitoring telesurgical procedures, detecting anomalies, and alerting surgeons to potential errors. ML algorithms can also analyze surgical outcomes to refine techniques and minimize risks. Cross-border telesurgery introduces additional layers of risk, particularly regarding technological failures. Issues such as latency, internet connectivity disruptions, and equipment malfunctions can have catastrophic consequences during a surgical procedure. For example, a sudden loss of communication between the surgeon and the robotic system may jeopardize the patient’s safety and lead to life-threatening complications. To mitigate these risks, healthcare providers must ensure robust technological systems with fail-safes and backups. One possible solution is to create an international regulatory body to oversee telesurgical standards and enforce stringent safety measures, particularly in LMICs, where healthcare infrastructure may be less developed. However, balancing innovation with patient safety requires careful ethical deliberation, as rushing untested technologies into practice can undermine trust in telesurgery and compromise patient welfare^[14–16]^. Professional accountability is another ethical consideration in cross-border telesurgery. In traditional surgical settings, the operating surgeon is physically present, making assigning responsibility for the procedure’s outcomes easier. In telesurgery, the distribution of roles among remote surgeons, local healthcare teams, and technical support staff creates ambiguity in accountability^[17]^. This raises important ethical questions: Who is ultimately responsible for the success or failure of the surgery??? How should liability be shared among the different parties involved??? Developing international legal frameworks that clarify liability distribution and mandate malpractice insurance for telesurgical providers could help address these concerns, ensuring patients are not left without recourse in malpractice or negligence cases^[6,7]^. The issue of trust is central to the ethical practice of cross-border telesurgery. Patients must have confidence not only in the competence of the remote surgeon but also in the reliability of the technology being used. Building this trust requires transparency in communicating the capabilities and limitations of the telesurgical system and the potential risks involved. Furthermore, patients may feel uneasy about being operated on by a surgeon who is not physically present, mainly if there is a lack of familiarity with the concept of telesurgery. Addressing these concerns requires open communication and a commitment to ethical principles, prioritizing the patient’s sense of security and trust^[8,9]^. In addition to patient-focused concerns, cross-border telesurgery poses ethical challenges for healthcare providers. For instance, surgeons may face conflicts of interest when performing procedures in countries with lower regulatory standards or when financial incentives drive decisions about patient care. These conflicts can compromise the integrity of medical practice and lead to suboptimal patient outcomes. To prevent unethical practices, national health authorities should establish guidelines regulating financial incentives in cross-border telesurgery and mandate disclosing potential conflicts of interest by participating surgeons^[10,11]^. Finally, data privacy and security are critical ethical considerations in cross-border telesurgery. The transmission of patient data across international borders raises concerns about compliance with privacy regulations such as the GDPR) in Europe or the HIPAA in the United States. Breaches of patient confidentiality can have severe consequences, including identity theft and stigmatization, particularly when sensitive medical information is involved. Harmonizing global data protection laws and enforcing strict cybersecurity protocols in telesurgical systems will safeguard patient information and maintain trust in cross-border telesurgical procedures. For example, by integrating AI-driven cybersecurity measures and blockchain-based encryption, healthcare institutions can reinforce the protection of patient data, ensuring compliance with global privacy regulations while maintaining trust in telesurgical procedures. Ensuring robust encryption and secure data handling protocols is essential to protecting patient privacy and maintaining trust in telesurgical processes^[12]^.

## Technological challenges and ethical implications

**Figure 2.**
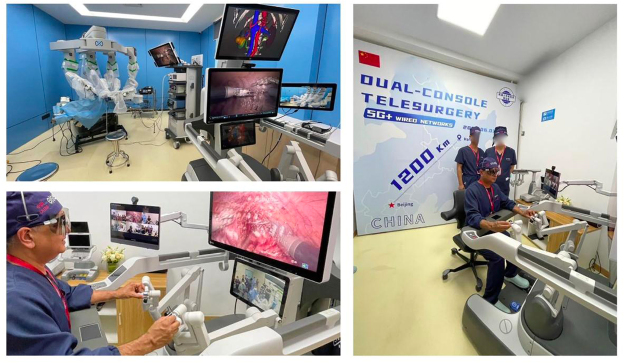
Illustration of a cross-border telesurgical setup. The surgeon operates remotely using a console, while the patient undergoes surgery via robotic arms. The system is connected through a high-speed 5G wired network (1200 km). Control signals: Commands from the surgeon are transmitted to the robotic arms. Real-time video feed: The surgeon’s screen displays live visuals from the patient’s body. Data transmission: Continuous exchange of information ensures precision and responsiveness – source: https://www.fightingprostatecancer.org/blog/2024/2/12/pr35oyplc56su0k7gpl1xm5jkq92oj.

The primary technological challenge in cross-border telesurgery lies in the reliance on high-quality communication networks. Telesurgery typically requires robust internet connections to support real-time, high-resolution video feeds and enable precise control over robotic surgical instruments (see Fig. 2)^[19]^. These instruments must be responsive to a surgeon’s commands with minimal latency, ensuring operations are carried out accurately. In some parts of the world, especially in rural or underserved regions, the infrastructure required to support such high-performance communications is not yet available, thus limiting the potential for equitable access to telesurgical procedures. The technological gap between regions with advanced infrastructure and those without could lead to disparities in the availability of medical services, undermining the benefits of telesurgery for global health^[20]^. Latency issues pose another significant concern in cross-border telesurgery. Even minor delays in communication can have devastating effects during complex surgeries. Surgeons rely on precise timing when controlling robotic surgical arms or performing delicate procedures, and any delay – however brief – can lead to unintended consequences. In a scenario where a surgeon operates remotely on a patient thousands of miles away, a few milliseconds of delay in transmitting data between the surgeon and the robotic system could affect the surgical outcome^[21]^. This risk is heightened in emergencies where rapid responses are crucial, and even small technological failures can compromise patient safety. As technology advances, addressing latency issues through infrastructure improvements, including 5G networks and low-latency communication systems, becomes imperative to mitigate such risks and ensure the reliability of telesurgical procedures^[2]^. The accuracy and reliability of robotic systems also present significant technological hurdles. While robotic systems are designed to enhance surgical precision, they are not infallible. These systems depend on a combination of AI, sensors, and ML algorithms to replicate the movements of a human surgeon. However, the limitations of these technologies must be considered. The accuracy of AI algorithms can be affected by the quality of the data they are trained on, which can vary significantly across regions, especially in areas with limited access to large, diverse datasets^[22–24]^. Inaccuracies in the AI-driven surgical system could lead to errors that may not be immediately detectable, putting patients at risk. Moreover, the evolution of robotic technology presents ethical dilemmas as decisions about the responsibility for errors become increasingly complex. If a robotic system malfunctions during surgery, it raises the question of who should be held accountable: the technology manufacturer, the surgeon operating the system, or the institution facilitating the procedure? These questions remain largely unresolved in many legal frameworks and underscore the need for clear guidelines on accountability in telesurgery^[3]^. The reliability of telecommunication systems is another crucial aspect of telesurgery that must be addressed to ensure patient safety. Interruptions in communication, such as power failures, server crashes, or network outages, can have disastrous consequences during a surgical procedure. A surgeon may lose real-time access to the patient’s vitals, the visual feed from inside the body, or the robotic system’s controls, leading to potentially fatal outcomes^[25–27]^. While advanced systems are being developed to address these issues, the reality is that telecommunication systems, particularly in less-developed countries, are not always as reliable as they need to be for safe telesurgery to occur. For instance, in rural areas where the infrastructure is not as developed, frequent power cuts or unstable internet connections could interrupt telesurgical procedures, exposing patients to unnecessary risks. Telesurgery can potentially reduce healthcare disparities by making specialized surgeries available to underserved populations. However, if access to this technology is not carefully managed, it could exacerbate existing inequalities and further marginalize vulnerable populations. Ethical frameworks must be developed to ensure that telesurgery benefits are distributed equitably and those in lower-income or remote regions are not left behind^[6]^. In addition to these concerns, there are ethical issues surrounding telesurgical procedures’ safety and effectiveness. While telesurgery has the potential to improve surgical outcomes by providing access to highly specialized expertise, it is essential to question whether it is appropriate to use this technology for all types of surgery. Some procedures may require more hands-on involvement from the surgeon, and in these cases, the risks associated with remote surgery may outweigh the benefits. For example, certain high-risk surgeries may require immediate intervention and adjustments that are difficult to execute remotely, leading to the potential for severe complications^[28]^. The decision to perform surgery remotely must be carefully considered, considering the nature of the procedure, the surgeon’s experience, and the availability of backup support if complications arise. Surgeons must adhere to strict ethical guidelines to ensure that telesurgery is only employed when it is in the patient’s best interest and when the technology is deemed reliable enough to provide a safe outcome^[7]^. The ethical responsibility of healthcare providers is another critical consideration in cross-border telesurgery. Surgeons and medical institutions must ensure they have the qualifications, training, and resources to perform telesurgical procedures competently. This includes ensuring all medical staff are well-versed in technology and prepared to handle potential complications during a remote procedure. Hospitals and surgical centers must also invest in the appropriate infrastructure to support telesurgery, including backup systems, training programs, and emergency protocols. Failing to do so can compromise patient safety and violate ethical standards of care^[8]^.

## Regulatory frameworks

To begin with, one of the foremost issues in cross-border telesurgery is jurisdiction. Jurisdiction determines which legal system governs telesurgical procedures, and conflicts may arise when the patient and the surgeon are in different countries. In traditional medical settings, jurisdictional issues are relatively straightforward, as a patient is typically treated within the same country as the medical provider. However, in telesurgery, the patient and the surgeon may be in different countries, potentially creating a conflict of laws. This scenario presents difficulties in determining which country’s laws apply if something goes wrong^[4–6]^. For instance, should a dispute arise over patient consent, liability, or data security, it remains unclear whether the legal framework of the patient’s country or the surgeon’s jurisdiction should take precedence. Jurisdictional conflicts arise when different legal systems govern the patient’s legal rights and the surgeon’s legal responsibilities. In such cases, it is crucial to establish protocols for determining which laws should govern the procedure and the enforcement of those laws if a malpractice claim arises. While some countries, such as the United States, have taken significant steps to address these issues by guiding the jurisdictional application of laws in telemedicine and telesurgery, many others have not yet created specific regulations for such procedures. The International Medical Informatics Association (IMIA) has called for international cooperation to address the challenges of jurisdiction in telesurgery, suggesting that governments and international bodies should collaborate to create common legal standards for cross-border medical procedures. One approach to resolving these legal inconsistencies involves the development of bilateral or multilateral agreements between countries, specifying the applicable legal frameworks for telesurgery. International treaties, such as the Convention on Cybercrime (2001), which addresses the issue of cross-border electronic crimes, can serve as a model for international agreements related to telesurgery (see Table 1)^[14–16]^. Such treaties could facilitate a unified approach to jurisdiction in telesurgery, making it easier to resolve legal conflicts and ensure that patients receive safe and legally compliant care, regardless of where they are located. Another critical aspect of the regulatory framework for cross-border telesurgery is licensing and accreditation. The World Health Organization (WHO) emphasizes international collaboration to develop standards and accreditation systems for telemedicine and telesurgery. The challenge here lies in the differences in medical licensing requirements across countries. A surgeon licensed to practice in one country may not be recognized in another, which could prevent the practice of telesurgery in a cross-border setting. For instance, the FSMB in the United States has established guidelines for telemedicine, including specific licensing requirements for telemedicine practitioners^[17–19]^. However, many countries do not have similar systems, which raises concerns about the qualifications and expertise of surgeons performing telesurgery remotely. Some countries have explored mutual recognition agreements to ensure compliance with different licensing requirements, allowing streamlined licensing approvals across jurisdictions. This could facilitate cross-border telesurgical procedures while maintaining high medical standards. In a cross-border context, patients may not clearly understand whether a surgeon is appropriately licensed and accredited to perform telesurgery in their jurisdiction. The licensing issue is compounded by the lack of standardized procedures for accredited healthcare facilities that offer telesurgery. Some countries have regulatory bodies that oversee the accreditation of hospitals and medical centers that provide telemedicine services, but these systems are not always compatible with those of other countries. As a result, hospitals that offer telesurgery may be subject to different standards depending on their location, which could affect the quality of care provided to patients^[20,21]^. An internationally recognized accreditation body, modeled after the JCI, could provide uniform licensing and accreditation standards for telesurgery. Liability and malpractice also present significant regulatory challenges in cross-border telesurgery. In traditional medical settings, determining liability in malpractice cases is relatively straightforward, as the healthcare provider and patient are in the same jurisdiction. However, when telesurgery is performed across borders, determining liability becomes much more complex^[4,5]^. Suppose a patient experiences harm during a telesurgical procedure. In that case, it may be challenging to determine whether the surgeon or the healthcare facility is responsible and which country’s laws should govern the malpractice claim. Furthermore, there may be disputes over which legal system should handle the claim, mainly if the surgeon and patient are in different countries. Many countries have adopted telemedicine malpractice insurance policies to address these challenges and cover the risks associated with remote medical procedures. For example, telesurgery surgeons in the United States must often carry telemedicine-specific malpractice insurance covering remote care risks. However, such insurance policies may not be recognized in other countries, making it difficult for surgeons to provide cross-border telesurgery services^[3,5,8]^. To address this gap, international agreements on malpractice insurance could be introduced, ensuring that patients and surgeons are adequately covered regardless of jurisdiction. International agreements on liability insurance and malpractice claims could help mitigate these issues and ensure that patients are adequately protected, regardless of where the surgery occurs. The issue of data protection and privacy is another significant regulatory concern in cross-border telesurgery. Compliance with international data protection laws is crucial given the sensitive nature of telesurgery data, including real-time surgical feeds, electronic health records, and patient biometrics. Telesurgery relies heavily on transmitting sensitive patient data, including medical images, patient histories, and real-time surgical information. The protection of this data is critical, particularly when it crosses international borders^[9–11]^. Different countries have different standards for data protection, which can create gaps in security when patient data are transmitted between jurisdictions. For instance, the GDPR in the European Union sets strict rules on how patient data should be handled. Still, other countries, such as the United States, may have less stringent regulations. While the HIPAA in the United States and the GDPR in the European Union aim to safeguard patient data, they differ in scope and application. GDPR requires explicit patient consent for data transfer, while HIPAA allows data sharing under specific circumstances without direct consent. To ensure compliance across jurisdictions, telesurgical platforms should implement robust encryption methods, cross-border data-sharing agreements, and patient consent protocols that align with GDPR, HIPAA, and other national regulations. If telesurgery involves patients from multiple countries, ensuring that data are protected in compliance with all relevant laws becomes challenging. International standards for data protection in telesurgery must be developed to address these concerns. These standards should establish clear guidelines on how patient data should be transmitted, stored, and accessed and measures to protect it from unauthorized access or breaches. Additionally, countries should work together to create uniform regulations on data protection, ensuring that patient privacy is upheld, regardless of where the data are transmitted. The HIPAA in the United States and the GDPR in the European Union provide valuable models for creating international data protection frameworks for telesurgery^[14,15,17]^. Several international organizations are working to establish regulatory frameworks for cross-border telesurgery. For example, the International Telecommunication Union (ITU) has set international standards for telemedicine and telesurgery. The WHO has also advocated for creating global guidelines for telemedicine, including telesurgery, that would address licensing, accreditation, and liability issues. However, creating such frameworks is complex and requires the collaboration of governments, healthcare providers, regulatory bodies, and other stakeholders^[1,19]^.

**Table 1:** Comparison of telesurgical regulatory frameworks: United States, European Union, and Asia

Regulatory aspect	United States (U.S.)	European Union (EU)	Asia (general trends)	Licensing requirements	Data privacy laws	Liability and malpractice	Cross-border challenges	Standardization of technology	Future considerations
Licensure and accreditation	Requires U.S. medical license + state-specific licensing	Mutual recognition in some EU countries, but national differences exist	Highly fragmented; varies widely by country	State-based licensing adds complexity	GDPR-compliant requirements across EU	Liability varies by country, often under EU directives	Coordination across multiple nations is difficult	EU has stricter medical device regulations	Push for more uniform accreditation
Telemedicine regulation	Governed by FDA, CMS, and state laws	EU Telemedicine Policy Framework (varies by nation)	Policies differ by country; some allow telesurgery, others restrict it	Limited national telemedicine policies	Data localization rules in some Asian nations	Weak legal protections in some countries	Lack of clear regional guidelines	Some countries rely on foreign-developed technology	Calls for regional coordination
Data protection and privacy	HIPAA-compliant, strict on patient data	GDPR ensures patient data protection	Varies: Some countries follow GDPR-like rules, others have lax laws	Strict U.S. encryption and security rules	EU-wide data protection harmonization	Stronger patient rights under GDPR	Variability in Asia complicates international cases	Asia increasingly aligning with global standards	Future AI-driven compliance measures
Malpractice and liability	Medical liability laws vary by state, high malpractice costs	EU-level patient protection laws, but enforcement is national	Often lacks well-defined malpractice laws for telesurgery	Complex malpractice laws create hurdles	GDPR mandates data breach notifications	Legal risks depend on national rules	Some Asian regions lack clear legal recourse	Push for uniform legal frameworks	AI-driven malpractice assessment being explored
Cross-border telesurgery	Challenging due to state and federal discrepancies	Easier within EU but still country-dependent	Largely unregulated in many countries	Federal-state mismatches complicate approvals	Strict EU-wide consent standards	Liability falls under national legal frameworks	Asia lags in formalizing telesurgery laws	Push for global legal harmonization	WHO and international bodies urging standardization
Reimbursement and insurance	Medicare/Medicaid provide limited coverage for telemedicine	EU countries have varying reimbursement policies	Most Asian nations lack telesurgery reimbursement	Insurance challenges limit adoption	EU working on unified coverage models	Medical tourism impact on costs	Lack of clear insurance models in Asia	Some nations piloting insurance coverage	More global insurers exploring telesurgery policies
Medical ethics and patient consent	Requires explicit consent under HIPAA	GDPR mandates strict consent protocols	Patient consent laws vary widely	Legal requirements differ by jurisdiction	Standardized EU-wide consent procedures	Ethical dilemmas in patient autonomy	Complex translations and cultural challenges	Ethical review boards help ensure compliance	AI-assisted consent tools being explored
Technology and interoperability	Regulated by the FDA, strict cybersecurity protocols	EU MDR regulates medical devices and software	Rapid tech adoption but regulatory gaps remain	Standardization issues for robotic surgery	EU-wide device approval system	Safety protocols still evolving	Language barriers & tech standards differ	Asia moving toward stricter safety regulations	AI integration in telesurgical systems
Future trends and challenges	AI-assisted telesurgery regulation still developing	Push for unified EU-wide telesurgery laws	Asia gradually implementing telemedicine laws	Stricter AI regulation is expected	GDPR compliance influences global trends	Liability and accountability in AI-driven surgery	International cooperation remains a challenge	WHO-led discussions on global telesurgery standards	AI, robotics, and blockchain expected to shape policies

While the United States follows a state-based licensure model with strict HIPAA compliance, the EU operates under GDPR with varying national regulations. Asia lacks uniform telesurgery laws, with some countries aligning with global standards while others remain underregulated. Liability, reimbursement, and insurance policies differ widely, complicating international telesurgical practices. Future trends indicate a push for AI integration, standardization of global frameworks, and improved cross-border collaboration – source: Authors’ Creations.

## Cybersecurity risks in telesurgery and risk mitigation strategies

One of telesurgery’s most pressing cybersecurity risk is unauthorized access to sensitive patient data. Patient records, surgical plans, and live procedural data are transmitted across networks, making them vulnerable to interception by malicious actors. Cybercriminals may exploit weak encryption standards or outdated security protocols to access confidential patient information, leading to data breaches and violations of privacy laws such as the GDPR in Europe and the HIPAA in the United States. Unauthorized access to patient data could result in identity theft, insurance fraud, and compromised medical histories, ultimately undermining patient trust in telesurgical procedures. Strong encryption protocols, such as Advanced Encryption Standard-256, should be implemented to protect data in transit and at rest, ensuring that only authorized personnel can access critical medical information^[1,2]^. Another significant cybersecurity risk in telesurgery is the potential for remote hacking of surgical robots. Telesurgical systems operate through real-time communication between a remote surgeon and a robotic interface, requiring seamless connectivity. However, if an attacker gains unauthorized access to the communication channel, they could manipulate robotic movements, disrupt procedures, or cause intentional harm to patients. Such cyber-physical attacks could have life-threatening consequences, making implementing robust authentication mechanisms and network security protocols imperative. Multifactor authentication, biometric verification, and blockchain-based security frameworks can enhance access control and prevent unauthorized interventions. Additionally, network segmentation can minimize exposure by isolating critical telesurgical systems from external threats, reducing the likelihood of cyber intrusions^[3,4]^. Denial-of-Service and Distributed Denial-of-Service (DDoS) attacks pose another major threat to telesurgery. These attacks overwhelm a network or server with excessive traffic, causing latency issues, delayed responses, or system crashes. In telesurgery, even a slight delay in response time can compromise procedural accuracy, potentially leading to severe surgical errors. Attackers may target hospitals or surgical centers with DDoS attacks to demand ransom payments, disrupt services, or create chaos in critical care settings. Implementing advanced intrusion detection and prevention systems can help identify and mitigate such attacks in real time. Additionally, redundant network pathways and failover mechanisms should be integrated to maintain connectivity and ensure continuous operation, even if one network component is compromised^[5,6]^. Another cybersecurity vulnerability in telesurgery is the risk of malware and ransomware infections. Malware, including viruses, worms, and spyware, can infiltrate telesurgical networks through phishing emails, malicious software updates, or compromised medical devices. Ransomware attacks, in particular, have become a significant concern for healthcare institutions, where attackers encrypt critical data and demand ransom payments for its release. If a telesurgical system is infected with ransomware, it could lead to procedural delays, system shutdowns, and loss of crucial patient data. Regular security updates, endpoint protection software, and network monitoring can reduce the risk of malware infections. Conducting cybersecurity training programs for medical professionals and IT staff can also help prevent phishing attacks and human errors contributing to security breaches^[7,8]^. Interoperability challenges further exacerbate cybersecurity risks in telesurgery. Modern telesurgical platforms integrate multiple technologies, including robotic-assisted systems, AI algorithms, cloud-based data storage, and 5G communication networks. While interoperability enhances efficiency, it also creates multiple points of vulnerability that hackers can exploit. Different medical devices and software systems may have varying security standards, leading to inconsistencies in protection levels. Standardizing security protocols across telesurgical devices and ensuring compliance with international cybersecurity frameworks can help mitigate these risks. For instance, the National Institute of Standards and Technology Cybersecurity Framework provides guidelines for securing healthcare technologies, including telesurgical systems^[9,10]^. Supply chain security is another crucial consideration in mitigating cybersecurity risks in telesurgery. Medical equipment manufacturers, software vendors, and third-party service providers are essential in developing and maintaining telesurgical systems. However, vulnerabilities in the supply chain can introduce security gaps that cybercriminals may exploit. Malicious actors may insert backdoors into software updates, tamper with medical device firmware, or compromise cloud-based storage solutions. Healthcare institutions should conduct thorough risk assessments of their vendors, implement stringent security requirements in procurement contracts, and ensure regular security audits of telesurgical equipment. Establishing a zero-trust security model – where access is granted based on strict verification rather than assumed trust – can further enhance the resilience of telesurgical infrastructure^[11,12]^. AI and ML are increasingly integrated into telesurgery for decision support, robotic automation, and predictive analytics. However, AI-driven telesurgical systems are not immune to cyber threats. Attackers can exploit vulnerabilities in AI algorithms through adversarial attacks, where small manipulations in input data can lead to incorrect surgical decisions. Additionally, AI models trained on biased or manipulated datasets may produce inaccurate results, compromising patient safety. Securing AI models through robust validation techniques, adversarial testing, and continuous monitoring can help safeguard telesurgical applications from malicious exploitation. Transparency in AI decision-making and regulatory oversight is essential to ensure AI’s ethical and safe use in telesurgery^[5,13]^. Cloud computing is critical in telesurgery by enabling remote data storage, real-time collaboration, and global accessibility. However, cloud-based telesurgical platforms introduce additional cybersecurity risks, including unauthorized data access, insider threats, and cloud service provider vulnerabilities. Implementing strong encryption mechanisms, multilayered authentication, and secure cloud configurations can mitigate these risks. Hospitals and healthcare organizations should establish clear data governance policies, ensuring compliance with regional and international data protection laws. Regular penetration testing and vulnerability assessments on cloud infrastructure can further enhance security resilience^[14,15]^. Governments and regulatory bodies must play an active role in enforcing cybersecurity standards for telesurgery. While existing regulations such as GDPR and HIPAA provide general guidelines for data protection, specific cybersecurity frameworks tailored to telesurgery are needed. An international collaboration between healthcare institutions, cybersecurity agencies, and medical device manufacturers can facilitate the development of global security standards. Cybersecurity incident response teams should be established to address potential breaches and coordinate rapid responses to emerging threats. Public-private partnerships can also foster innovation in cybersecurity technologies, driving the adoption of next-generation security solutions for telesurgical applications^[16,17]^.

## Lessons learned

Jurisdictional uncertainty is one of the most significant legal challenges of cross-border telesurgery. Telesurgery, by its nature, involves the participation of healthcare professionals and patients in different geographical locations, which raises questions about which laws and regulations apply to the procedure^[1–3]^. In traditional medical practice, a clear jurisdiction exists based on the location of the healthcare provider or the patient. However, in the case of telesurgery, where the surgeon may be located in one country and the patient in another, the situation becomes more complex. Legal disputes have arisen regarding the application of medical malpractice laws, where patients have sought redress for surgical errors that occurred during remote procedures^[3–5]^. Some jurisdictions have unclear provisions about whether a surgeon performing telesurgery in one country can be held accountable for the consequences of their actions in another country. The lack of clear legal frameworks for cross-border medical practice has highlighted the need for greater clarity in the telesurgery regulations, with some countries taking steps to update their medical licensure laws to address these issues. In addition to jurisdictional concerns, the licensing and accreditation of healthcare providers are also significant legal challenges in cross-border telesurgery. Countries have different medical licensure standards, and telesurgical procedures often involve professionals not licensed in the country where the patient resides. This raises credentialing issues, as the healthcare provider in the remote location may not be recognized as qualified to perform surgery in the patient’s country. This has the potential to undermine the integrity of the procedure and, in some cases, compromise patient safety^[6–8]^. Legal precedents in telesurgery cases have shown that there is no consistent global framework for accrediting telesurgeons or healthcare facilities, which makes it difficult for patients and healthcare institutions to determine whether the professionals involved in a telesurgical procedure are adequately trained and qualified. Countries with robust medical licensing systems, such as the United States and the European Union, are working to establish more explicit frameworks for recognizing foreign credentials in telesurgical contexts. However, these efforts are still in their infancy. Liability and malpractice are central to the ethical and legal challenges of telesurgery. The issue of who is responsible when something goes wrong during a telesurgical procedure is one of the most complex aspects of this practice. Legal cases involving telesurgery malpractice have highlighted the difficulty in assigning accountability for errors. In some cases, the surgeon performing the surgery remotely has been held liable for mistakes made during the procedure. In contrast, in other cases, the blame has been placed on the healthcare facility hosting the patient or the technicians managing the telecommunication systems^[9–11]^. These legal disputes underscore the need for clear protocols to define the responsibilities of all parties involved in telesurgical procedures. One critical lesson learned is the importance of developing standardized legal agreements that outline the roles, responsibilities, and liabilities of all individuals and organizations involved in the procedure. Such agreements are essential for protecting healthcare providers and ensuring patients receive the highest standard of care. Malpractice insurance is another important consideration, as telesurgeons and healthcare institutions must ensure that their insurance policies cover cross-border procedures and potential legal disputes^[12,13]^. Data protection and privacy are also significant ethical and legal issues in telesurgery. The transmission of sensitive medical data, including patient records and real-time surgical information, over the Internet poses risks to patient confidentiality. While international standards such as the GDPR in the European Union and the HIPAA in the United States have set guidelines for protecting medical data, these regulations may not apply uniformly across countries. In the case of cross-border telesurgery, patient data may be transmitted through servers in different countries, each with its own laws governing data protection. This raises concerns about the potential for data breaches, unauthorized access to patient information, and the lack of accountability when breaches occur^[5,14,15]^. The lessons learned from legal cases involving data breaches in telesurgery have emphasized the need for stronger global regulations and more secure communication systems. Telesurgeons and healthcare institutions must adopt best practices in data security, including encrypted communication channels, secure servers, and robust data management systems, to mitigate the risk of privacy violations. Informed consent in telesurgery presents its own set of ethical and legal challenges. Obtaining informed consent in traditional surgery is a well-established process that involves providing patients with detailed information about the procedure, its risks, benefits, and alternatives. However, obtaining consent becomes more complicated in telesurgery, where the surgeon may be located remotely. Patients may feel uncomfortable or uncertain about having surgery performed by a surgeon who is not physically present in the exact location. Furthermore, patients may not fully understand the risks associated with telesurgery, mainly if they are not familiar with the technology being used^[16–18]^. Several legal cases have highlighted the importance of ensuring that patients understand what telesurgery entails and provide their consent willingly. Legal lessons learned from these cases suggest that it is essential for healthcare providers to take extra steps in explaining the telesurgical procedure, ensuring that patients are fully informed and not coerced into consent. Additionally, there must be clear documentation of the consent process, including confirmation that the patient understands the technology and any potential risks. The ethical challenges of cross-border telesurgery also include concerns about equity and accessibility. While telesurgery has the potential to provide high-quality care to patients in remote or underserved areas, there is a risk that it may exacerbate existing healthcare disparities. Wealthier countries and individuals are more likely to have access to advanced technologies and skilled surgeons capable of performing telesurgery. At the same time, poorer regions may lack the infrastructure or resources to implement such procedures^[19–21]^. Furthermore, social and cultural factors such as language barriers, lack of trust in remote medical procedures, and healthcare education disparities may affect telesurgery access. Ethical considerations around equity in telesurgery emphasize the need for inclusive policies that ensure all patients, regardless of their socioeconomic background, can benefit from these advancements. Lessons learned from ethical dilemmas in telesurgery have highlighted the need for global collaborations to ensure equitable access to telesurgical care, emphasizing affordable and culturally sensitive solutions for disadvantaged populations^[22]^. Patient safety and risk management are at the heart of the ethical concerns in telesurgery. While telesurgery offers many advantages, such as the ability to access expert surgical care from a distance, it also introduces risks associated with technological failures. These risks include equipment malfunctions, communication breakdowns, and delays in transmitting critical information^[23,24]^. The ethical question arises regarding whether the patient or the healthcare provider should bear these risks. Some patients may feel uncomfortable undergoing surgery remotely due to concerns about the reliability of the technology, while others may see telesurgery as a promising option for overcoming geographical barriers. Ethical lessons from telesurgical practice emphasize the importance of ensuring that patients are fully informed about the potential risks and that appropriate risk management strategies are in place. This includes regular maintenance of equipment, testing of communication systems, and having contingency plans for emergencies^[25–27]^. The lessons learned from legal and ethical challenges in cross-border telesurgery underscore the need for a comprehensive framework that addresses the multifaceted issues involved in this practice. Such a framework should integrate legal clarity, ethical principles, and technological safeguards to ensure telesurgery is conducted safely, equitably, and responsibly. Developing international telesurgery guidelines will ensure its long-term success and acceptance as the field evolves. The cross-border nature of telesurgery presents unique challenges, but it also offers significant opportunities to improve global healthcare delivery. By addressing the legal and ethical issues this practice raises, we can create a future where telesurgery is a technological advancement and a responsible, ethical, and equitable approach to patient care^[28,29]^.

## Future research directions and limitations

The increasing reliance on telecommunication technologies in healthcare necessitates the development of unified international regulatory frameworks that can guide the practice of telesurgery across borders. One of the significant challenges in cross-border telesurgery is the lack of a harmonized legal framework that can apply uniformly across different jurisdictions. Different countries have varying laws regarding medical licensure, patient consent, malpractice liability, and data privacy, which complicates the provision of telesurgical services on a global scale. This fragmentation of regulatory structures makes it difficult for healthcare providers and patients to navigate the legal complexities associated with telesurgery^[12,13]^. Furthermore, there is a lack of real-world case studies analyzing how these regulatory frameworks function in telesurgical procedures across different jurisdictions. Future research should focus on empirical studies evaluating patient outcomes, legal disputes, and compliance challenges in international telesurgery settings. The future of telesurgery will require establishing a set of universally accepted guidelines and legal frameworks that can be applied across borders to mitigate these challenges. An essential step toward this goal will involve the collaboration of international regulatory bodies such as the WHO and the International Medical Association. These organizations will play a critical role in coordinating efforts to create global standards that govern the practice of telesurgery. By developing guidelines that address issues such as licensing, credentialing, and liability, these bodies can ensure that telesurgery practices adhere to established norms of medical ethics and safety, irrespective of the country in which they are conducted. Furthermore, these global guidelines should be flexible enough for individual countries’ unique legal and cultural contexts while ensuring patient safety and rights are not compromised. For instance, countries with stricter data protection laws could adopt tailored approaches that align with international standards while preserving their legal frameworks on privacy and data security^[5,7,10]^. Another critical area of focus is the improvement of patient consent processes. Obtaining informed consent is essential to medical practice, ensuring patients are fully aware of the risks, benefits, and alternatives to any medical intervention. In the context of cross-border telesurgery, the process of obtaining informed consent becomes more complex due to language barriers, cultural differences, and the physical separation between the surgeon and the patient^[11–13]^. To address these challenges, future telesurgical practices must employ advanced technologies such as real-time translation tools and culturally sensitive consent protocols to ensure that patients understand the nature of the procedure and its associated risks. Additionally, it will be necessary to design consent forms that are not only legally valid in multiple jurisdictions but also provide clear and accessible information to patients in a manner that respects their autonomy. Informed consent in telesurgery also raises important ethical questions about autonomy and trust. The physical absence of the surgeon during the procedure and the reliance on technology for performing complex operations may raise concerns among patients regarding the effectiveness of the intervention and the qualifications of the surgical team^[5,14,15]^. These concerns could undermine trust in the telesurgical process and diminish patient autonomy. To counter these concerns, telesurgery practices must prioritize transparency and establish robust communication channels between the patient and the surgical team. Surgeons should engage patients in discussions about the technology involved, the qualifications of the surgical team, and the steps taken to ensure patient safety. These conversations should be ongoing, allowing patients to voice their concerns and make informed decisions about their participation in telesurgery. Equity and accessibility will also be central to the future development of telesurgery. As the technology becomes more widely available, there is a risk that access to telesurgical procedures may be limited to certain populations, especially high-income countries or urban areas, while leaving underserved populations in low-income regions without access to these life-saving interventions^[16,17]^. The digital divide, including disparities in access to the Internet, mobile technologies, and healthcare infrastructure, will need to be addressed to ensure that telesurgery can be accessible to all patients, regardless of their geographic location or socioeconomic status. Collaborative efforts between governments, healthcare providers, and technology companies will be crucial in bridging this gap. Investment in rural and underserved areas, the development of low-cost telecommunication solutions, and the establishment of telemedicine infrastructure in resource-limited settings can enhance the accessibility of telesurgery, ensuring that its benefits are equitably distributed^[18,19]^. Moreover, technological advancements in telesurgery will likely continue rapidly, opening up new possibilities for the practice and increasing its precision and safety. However, these advances must be accompanied by robust ethical frameworks that can address the potential risks associated with the increasing reliance on technology in surgery. Future telesurgical systems must be designed with the highest safety and reliability standards, incorporating redundancies and fail-safes to minimize the risk of technical failure^[20,21]^. Surgeons and healthcare providers must be adequately trained to use these advanced systems, and rigorous testing protocols should be established to ensure that the equipment meets the necessary safety standards. Additionally, more research is needed to explore how countries outside the United States and Europe manage telesurgical data privacy. A comparative study of data protection laws in emerging markets, where telemedicine adoption is rising, would provide a more globally inclusive understanding. The future of telesurgery will also involve the development of AI and ML applications to assist in surgical decision-making. These technologies hold great promise in enhancing the precision of telesurgery by providing real-time data analysis, predictive modeling, and decision support. AI-powered systems could assist surgeons in making complex decisions during the procedure, improving the accuracy of diagnoses and optimizing patient outcomes^[22,23]^. However, the integration of AI in telesurgery raises significant ethical concerns regarding the role of machines in making decisions that directly affect patient health. Ethical questions related to accountability, transparency, and the delegation of decision-making to machines will need to be addressed to ensure that AI is used responsibly and in a manner that respects patient autonomy and rights. Regulatory bodies must develop standards for using AI in telesurgery, ensuring these systems are subject to appropriate oversight and scrutiny. The role of patient safety and risk management in telesurgery cannot be overstated. As the technology continues to evolve, the potential for unexpected complications and adverse outcomes remains a concern. Future telesurgical practices must incorporate comprehensive risk management strategies that consider the unique challenges of remote procedures^[24,25]^. These strategies should include preoperative assessments, continuous monitoring during the procedure, and postoperative follow-up to ensure that any complications are promptly addressed. Surgeons and healthcare providers should also be prepared for technical failures or human error, and clear protocols for managing such incidents should be in place. In addition, ethical guidelines will need to be developed to address patient safety challenges in a setting where the surgeon is not physically present^[26,27]^. As telesurgery evolves, more research is needed on the impact of emerging technologies such as AI-assisted procedures and robotic automation. Future studies should assess how these advancements influence liability, accreditation, and cross-border compliance in telesurgical practice. Finally, collaboration between healthcare professionals, governments, international organizations, and technology developers will be essential in shaping the future of telesurgery. Developing a global framework for telesurgery that addresses legal, ethical, technological, and regulatory challenges will require the collective efforts of all stakeholders. The role of international cooperation in advancing telesurgery cannot be overstated. Through collaborative efforts, countries can share knowledge, resources, and best practices, creating a more equitable and sustainable model for the global practice of telesurgery^[28–30]^.

## Concluding remarks

While cross-border telesurgical procedures hold immense promise in revolutionizing healthcare delivery, particularly in remote or underserved regions, they present significant legal and ethical challenges. Jurisdictional issues, licensing requirements, and the complexities of malpractice liability are just a few of the legal hurdles that must be addressed. Ethically, the concerns surrounding informed consent, patient safety, equity, and trust in technology must be carefully managed to ensure that telesurgery remains a beneficial and responsible practice. The rapid pace of technological advancements necessitates the establishment of comprehensive regulatory frameworks that can address these challenges across borders. Collaborative international efforts will be key to creating standardized protocols, ensuring patient safety, and maintaining the integrity of the medical profession. Global regulatory bodies such as the WHO, the ITU, and the IMIA could play pivotal roles in overseeing the development and enforcement of international telesurgical standards.

Additionally, organizations like the International Organization for Standardization could contribute by establishing technical and safety guidelines for telesurgical procedures. Ultimately, as telesurgery continues to evolve, it is essential to strike a balance between innovation and patient welfare, ensuring that the potential benefits of these procedures are realized without compromising patients’ rights, safety, or trust. Future efforts should focus on developing robust, globally recognized legal and ethical guidelines that foster innovation while safeguarding the interests of all stakeholders involved.

## Data Availability

This published article and its supplementary information files include all data generated or analyzed during this study.
